# High Expression of the Sd^a^ Synthase B4GALNT2 Associates with Good Prognosis and Attenuates Stemness in Colon Cancer

**DOI:** 10.3390/cells9040948

**Published:** 2020-04-11

**Authors:** Michela Pucci, Inês Gomes Ferreira, Martina Orlandani, Nadia Malagolini, Manuela Ferracin, Fabio Dall’Olio

**Affiliations:** Department of Experimental, Diagnostic and Specialty Medicine (DIMES), General Pathology Building, University of Bologna, Via San Giacomo 14, Via San Giacomo 14, 40126 Bologna, Italy; michela.pucci3@unibo.it (M.P.); ines.gomesferreira2@unibo.it (I.G.F.); martina.orlandani@unibo.it (M.O.); nadia.malagolini@unibo.it (N.M.); manuela.ferracin@unibo.it (M.F.)

**Keywords:** glycosylation, sugar antigens, glycosyltransferases, gene expression, stemness, microarray analysis

## Abstract

Background: The carbohydrate antigen Sd^a^ and its biosynthetic enzyme B4GALNT2 are highly expressed in normal colonic mucosa but are down-regulated to a variable degree in colon cancer tissues. Here, we investigated the clinical and biological importance of B4GALNT2 in colon cancer. Methods: Correlations of B4GALNT2 mRNA with clinical data were obtained from The Cancer Genome Atlas (TCGA) database; the phenotypic and transcriptomic changes induced by B4GALNT2 were studied in LS174T cells transfected with B4GALNT2 cDNA. Results: TCGA data indicate that patients with high B4GALNT2 expression in cancer tissues display longer survival than non-expressers. In LS174T cells, expression of B4GALNT2 did not affect the ability to heal a scratch wound or to form colonies in standard growth conditions but markedly reduced the growth in soft agar, the tridimensional (3D) growth as spheroids, and the number of cancer stem cells, indicating a specific effect of B4GALNT2 on the growth in poor adherence and stemness. On the transcriptome, B4GALNT2 induced the down-regulation of the stemness-associated gene *SOX2* and modulated gene expression towards an attenuation of the cancer phenotype. Conclusions: The level of B4GALNT2 can be proposed as a marker to identify higher- and lower-risk colorectal cancer patients.

## 1. Introduction

**Figure 1:**
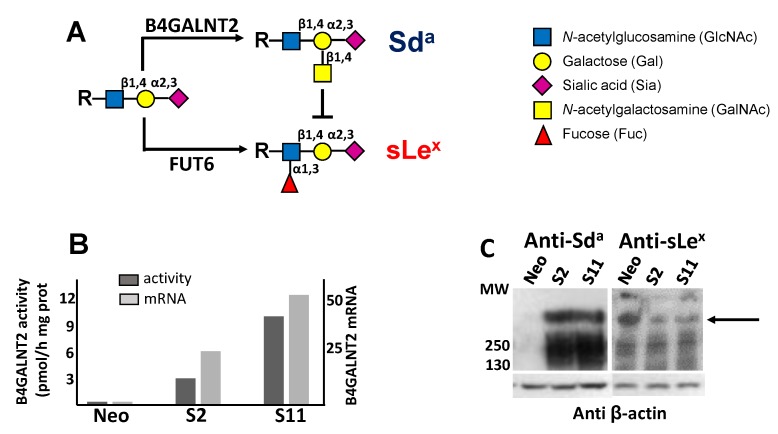
Biochemical characterization of B4GALNT2-transfected cell lines. (**A**) the Sd^a^ and the sLe^x^ antigens derive from alternative and mutually exclusive terminations of a common α2,3-sialylated type 2 structure. (**B**) both the enzymatic activity (dark gray) and the mRNA (light gray) of B4GALNT2 were negligible in Neo cells, but strongly expressed in clones S2 and S11 as detected by RT-PCR and normalized with β-actin. (**C**) Western blot analysis of Neo cells and of B4GALNT2-transfected clones with anti-Sd^a^ (left) and anti-sLe^x^ (right) antibodies, revealing a partial replacement of the sLe^x^ antigen with the Sd^a^ (arrow).

Colorectal cancer (CRC) remains a worldwide leading cause of cancer deaths, notwithstanding the improved efficacy of the available therapies. Key factors for the success of the therapy include an early diagnosis and approaches tailored to the risk profile of each patient, sparing invasive and expensive therapies to lower-risk patients. Glycosylation is a very frequent post-translational modification of proteins, which undergoes profound changes during neoplastic transformation [1,2]. The Sd^a^ carbohydrate antigen is composed of an α2,3-sialylated galactose residue to which an N-acetylgalactosamine residue is β1,4 linked (Siaα2,3(GalNAcβ1,4) Gal-R), where R is the underlying carbohydrate structure (Figure 1A). The last step of the Sd^a^ antigen biosynthesis is mediated by the enzyme β1,4N-acetylgalactosaminyltransferase 2 (B4GALNT2) [3], the product of the *B4GALNT2* gene [4,5,6]. Transcription of the *B4GALNT2* gene generates at least two different transcripts that only differ in the first exon. These two transcripts contain a translational start site, from which two different transmembrane polypeptides originate: one, referred to as “long form”, contains an unusually long cytoplasmic tail [5,6]; the second, known as “short form”, is provided with a cytoplasmic tail of conventional length [7]. Previous studies have shown a higher enzymatic activity of the short form compared with the long form [8]. The normal human colonic mucosa usually expresses very high levels of the B4GALNT2 mRNA and enzyme activity as well as high levels of the Sd^a^ antigen. On the contrary, in CRC tissues, both the B4GALNT2 [9,10] and the Sd^a^ antigen [8] are markedly down-regulated, although at a very variable level among patients. In fact, in the cancer tissues of the majority of the patients, B4GALNT2 is virtually undetectable, while in a minority, a quite high activity is detectable. Both normal and cancerous colonic tissues express mainly, if not exclusively, the short form of B4GALNT2. The α2,3 sialylated type 2 sugar chains on which B4GALNT2 elaborates the Sd^a^ antigen can also be utilized by fucosyltransferases (mainly fucosyltransferase 6 (FUT6)) for the biosynthesis of the cancer-associated antigen sialyl Lewis X (sLe^x^) [11,12,13]. Our group [8] and others [14] showed that the forced expression of B4GALNT2 in CRC cell lines partially replaces the sLe^x^ with the Sd^a^ antigen. In gastrointestinal cell lines, this modification has been shown to reduce the metastatic potential [14,15]. However, the clinical implications of B4GALNT2 expression in CRC have never been investigated. To obtain significant clinical correlations between gene expression and clinical parameters, it is necessary to access large cohorts of fully characterized patients, such as The Cancer Genome Atlas (TCGA), which contains gene expression and clinical data from hundreds of patients. Owing to the well-recognized importance of glycosylation in cancer, we used TCGA data to compare the prognostic predictive potential of several glycosyltransferases involved in the biosynthesis of cancer-associated glycans. In a preliminary survey of TCGA, we noticed that among various glycosyltransferases involved in colon cancer, B4GALNT2 displayed a very good predictive potential in that patients retaining higher levels of B4GALNT2 mRNA displayed a much longer overall survival. In particular, all long-time survivals displayed high levels of B4GALNT2 mRNA. To obtain insight into the mechanisms linking B4GALNT2/Sd^a^ expression to the CRC phenotype, we analyzed the phenotype and the transcriptome of LS174T cells transfected with the short form of B4GALNT2. We found that B4GALNT2 expression reduces the clonogenic ability in non-adherent conditions and the stemness of the cells through the modulation of the gene expression.

## 2. Materials and Methods.

### 2.1. Analysis of TCGA Database

Gene expression data and clinical information for 623 colorectal adenocarcinoma samples and 51 normal colonic tissues were downloaded from the TCGA database using the Firebrowse website [16]. RNA-Seq by Expectation Maximization (RSEM)-normalized data for the colon adenocarcinoma (COADREAD) cohort were matched with clinical data from the Clinical Pick Tier1 archive. B4GALNT2 mRNA expression was compared with stage, microsatellite stability (MS) status, response to treatment, histological type, and survival. Since the samples did not present a normal distribution of B4GALNT2 expression, non-parametric statistical tests were used. The Mann–Whitney test was used to analyze the difference of B4GALNT2 expression in normal and tumor tissues of mucinous vs. non-mucinous histological type. The Kruskal–Wallis test was used to evaluate B4GALNT2 mRNA expression across cancer stages and MSS/MSI groups. The survival curve was estimated by the Kaplan–Meier method, and the Mantel–Cox log-rank test was performed to test differences between the survival curves. Identification of highly expressed genes in the high and low B4GALNT2 expressers was performed through two-way ANOVA and Bonferroni’s multiple comparison test.

### 2.2. Cell Lines

LS174T(ATCCR^®^ Number: CL-188™) cell line was transfected with an expression vector for the short form of B4GALNT2 cDNA cloned in pcDNA3 or with the empty vector as detailed previously [8], generating the two B4GALNT2-expressing clones S2 and S11 and the polyclonal negative control Neo population, respectively. Cells were cultured in DMEM supplemented with 10% FBS and antibiotics in a humidified incubator with a 5% CO_2_ atmosphere at 37 °C. B4GALNT2 enzymatic activity was measured as the difference between the incorporation of radioactive GalNAc on fetuin and asialofetuin, as previously described [5]. B4GALNT2 mRNA was measured by real-time (RT) PCR as previously described [17]. Western blot analysis with an anti-Sd^a^ antibody KM694 and an anti-sLe^x^ antibody (CSLEX1) was performed as detailed elsewhere [8]. Cell lines were genotyped using highly polymorphic short tandem repeat loci, which were amplified using the PowerPlex^®^ 16 HS System (Promega). Fragment analysis was done on an ABI3730xl (Life Technologies), and the resulting data were analyzed with GeneMarker HID software (Softgenetics) by Microsynth (Switzerland). Reports are available on request.

### 2.3. Soft Agar Growth Assay

One milliliter of a 0.5% agar solution in complete DMEM was dispensed in each well of a six-well plate and allowed to solidify. On top of this layer of agar, 1 mL of a 0.3% agar solution in complete DMEM medium containing 1 × 10^4^ cells per well was dispensed in triplicate. The plates were incubated for two weeks at 37 °C in a humidified incubator. To evaluate the number of colonies formed, the plates were fixed and colored for one hour at room temperature with a solution containing formaldehyde (4%) and crystal violet (0.005%) in phosphate buffered saline (PBS, 20 mM phosphate buffer pH 7.5, 0.15 mM NaCl). Pictures were taken at 4X magnification, and colonies were counted. Statistical analysis was performed using the non-parametric Kolmogorov–Smirnov test.

### 2.4. Tridimensional (3D) Culture

Cells were seeded in six-well plates whose bottoms were coated with 0.5% agar in complete DMEM. Spheroid growth was monitored every 2–3 days. Owing to their non-adherent condition, it was impossible to quantitate spheroids by counting. Thus, cells were quantitatively collected and homogenized, and the protein concentration, as well as the volume of each homogenate, was measured. The number of cells was calculated using the protein concentration of a homogenate obtained from a known number of cells grown in standard conditions as a reference.

### 2.5. Wound-Healing Assay

The wound-healing assay was performed using Culture-Insert 2 Well (Ibidi). Aliquots of 5 × 10^4^ cells were seeded in each well. When the cells reached confluency, the insert was removed and the healing of the wound was measured by taking pictures at 4× magnification. The area free of cells was measured using the MRI Wound Healing Tool of ImageJ [18]. The statistical analysis was performed using two-way ANOVA and Tukey’s multiple comparisons test.

### 2.6. ALDEFLUOR Assay

ALDEFLUOR (Stem Cell Technologies) was activated following the manufacturer’s instructions and added to 5 × 10^5^ aliquots of cells. Half of the cell suspension was treated with DEAB, a specific ALDH inhibitor used as a negative control. After 45 min at 37 °C, cells were washed and suspended in ALDEFLUOR buffer. The fluorescent signal was acquired with a FACSCalibur flow cytometer and Cell Quest Pro software. On a dot plot with FL1 (green fluorescence) on the X axis and side scatter (SSC) on the Y axis, we set the fluorescence of the DEAB sample (negative control) and defined the area for ALDH-positive cells. Cells included in this area were considered ALDEFLUOR-positive.

### 2.7. Transcriptomic Analysis

Transcriptomic analysis of RNA from LS174T Neo and S2/S11 cells grown either in standard 2D conditions or in 3D conditions (as spheroids) was performed in duplicate using Agilent whole human genome oligo microarray (G4851A) as previously described [19]. Statistical analysis was performed using a moderated *t*-test, and the false discovery rate was controlled with the multiple testing correction Benjamini–Hochberg with Q = 0.05. Pathway analysis of differentially expressed genes was determined using the web-based software MetaCore (GeneGo, Thomson Reuters). Gene function was studied through an extensive literature search.

## 3. Results

### 3.1. Survey of the TCGA Database

**Figure 2:**
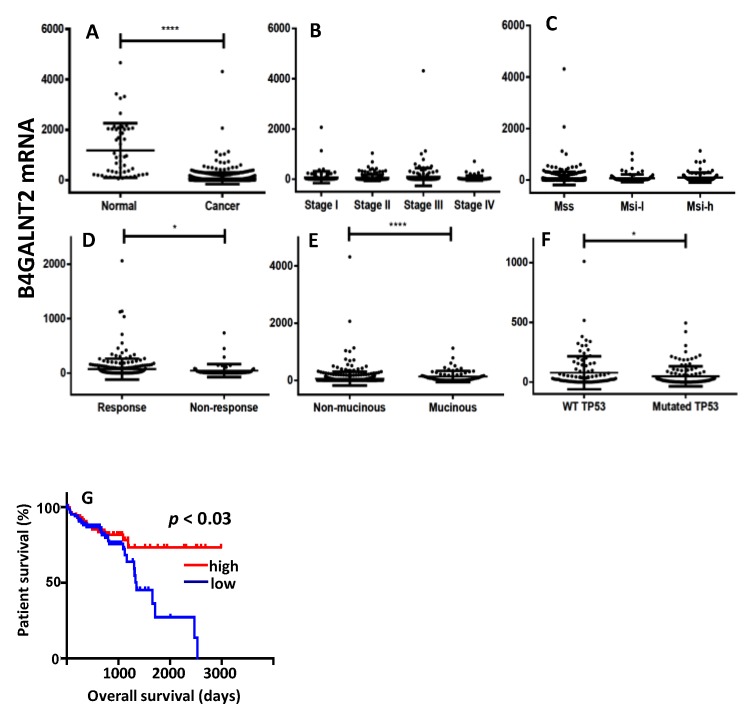
The Cancer Genome Atlas (TCGA) data. (**A**) Expression level of B4GALNT2 mRNA in normal mucosa and colorectal cancer (CRC) specimens. (**B**–**F**) Expression of B4GALNT2 mRNA in CRC specimens grouped according to stage (**B**), microsatellite stability status (**C**), response to therapy (**D**) subtypes (**E**), and TP53 mutation (**F**). (**G**) Kaplan–Meier survival curves of patients grouped in the groups of high expressers (15th upper percentile, red) or no expressers (15th lower percentile, blu) of mRNA B4GALNT2 expression. MSS: microsatellite stable; MSI-l: microsatellite instable-low; MSI-h: microsatellite instable-high. * *p* ≤ 0.05; **** *p* ≤ 0.0001.

The relationship between *B4GALNT2* gene expression and clinical parameters of CRC and normal specimens was investigated in the transcriptomic data from the TCGA database. As shown in Figure 2A, the mean level of B4GALNT2 mRNA in CRC tissues was much lower than in normal tissues, albeit extremely variable. No significant association existed between B4GALNT2 expression and stage or microsatellite stability status (Figure 2B,C). However, B4GALNT2 expression was significantly higher in the groups of therapy responder (Figure 2D), non-mucinous subtype (Figure 2E), and wild-type *TP53* (Figure 2F). On the other hand, little or no relationship was observed with *KRAS*, *BRAF*, and *APC* mutations (data not shown). The most striking effect of the level of B4GALNT2 expression was observed on the overall survival (Figure 2G). Patients falling in the lower 15th percentile (all lacking detectable B4GALNT2 mRNA) and those falling in the higher 15th percentile displayed very similar survival curves within the first 1000 days, while long-term survivals belonged exclusively to the high-B4GALNT2-expressers group (Figure 2G).

The cohorts of high and low B4GALNT2 expressers displayed very different gene expression signatures. In fact, samples from the upper percentile showed up-regulation of several genes (Table 1), five of which (*CLCA1*, *FCGBP, MUC2, MUC5B, AGR2*) were related to the formation of the mucous layer. Three genes were involved in the immune function (*LCN2, IGJ, PIGR*), particularly in the formation and transport of polymeric immunoglobulins, while three genes (*REG1A, REG4, TFF3*) were involved in repair and maintenance of the epithelial layer. On the contrary, the growth-promoting gene *IGF2* displayed up-regulation in low B4GALNT2 expressers. Altogether, these data support a view in which high B4GALNT2 expressers displayed up-regulation of genes involved in functions associated with a normal epithelium (mucus formation, immunoglobulin secretion, and epithelial integrity), while low B4GALNT2 expressers were associated with the overexpression of a growth-promoting gene.

### 3.2. Phenotypic Impact of B4GALNT2 Expression on Colon Cancer Cells

Owing to the clear association between high B4GALNT2 and better prognosis, the impact of B4GALNT2 expression on the malignant phenotype of a colon cancer cell line was studied using, as a model, LS174T cells transfected with the short form of B4GALNT2 or mock-transfected [8]. The three cell lines analyzed were Neo—a polyclonal population of mock-transfectants—and S2 and S11, two B4GALNT2-transfected clones. As shown in Figure 1B, the level of B4GALNT2 mRNA and enzyme activity in mock transfectants was nearly undetectable, while it was high in S2 and S11 clones. In S2 and S11 clones, but not in Neo cells, the Sd^a^ antigen was strongly expressed on high-molecular-weight proteins. On the other hand, the sLe^x^ antigen, which is also carried by high-molecular-weight proteins, was more strongly expressed by Neo cells than by S2 and S11 clones (Figure 1C). This is due to the previously documented competition between the fucosyltransferases synthesizing sLe^x^ and B4GALNT2 [8,14] (Figure 1A).

**Figure 3:**
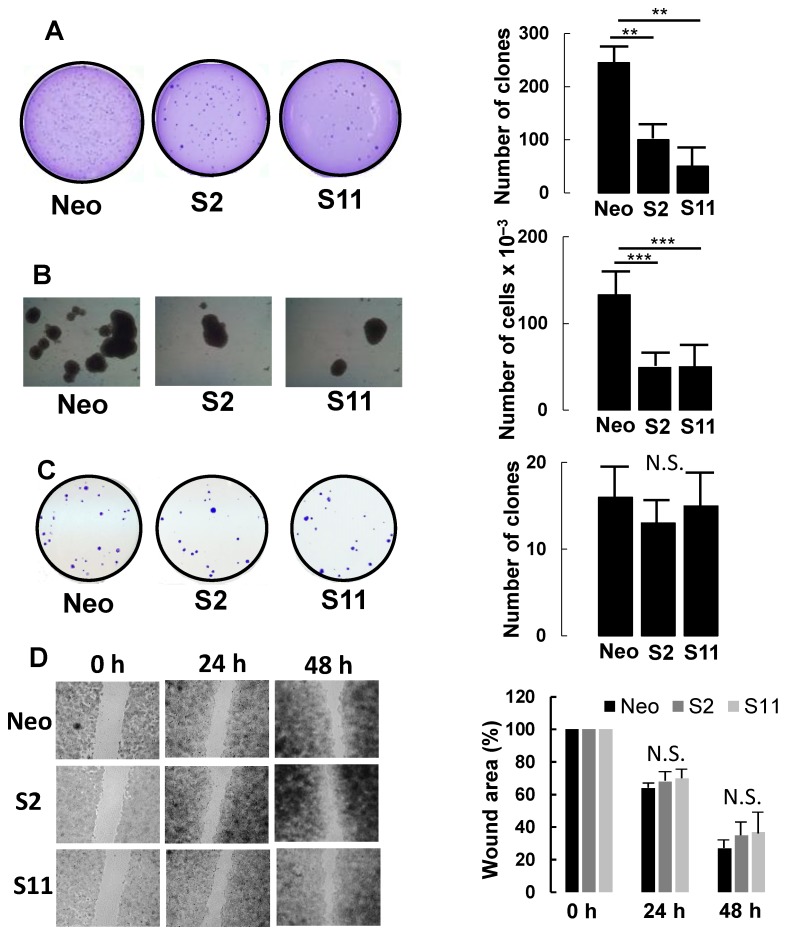
Phenotypic characterization of B4GALNT2-expressing cells and mock-transfectants. (**A**) growth in 0.33% agar. (**B**) tridimensional growth as spheroids. (**C**) clone formation in standard growth conditions. (**D**) wound healing assay. Experimental details are provided in Materials and Methods. ** *p* ≤ 0.01, *** *p* ≤ 0.001, N.S. = Not significant.

Compared with mock-transfected Neo cells, both S2 and S11 clones displayed a strongly reduced ability to grow in a semi-solid medium (soft agar) (Figure 3A), forming 20–40% of the clones formed by Neo cells.

B4GALNT2-expressing clones also displayed a 60% reduction in ability to grow as spheroids in a completely liquid medium (Figure 3B). Interestingly, the decreased clonogenic capability was evident only in conditions of poor or no adhesion. Indeed, when a small number of Neo, S2, or S11 cells were seeded in standard conditions, the number of growing colonies was similar (Figure 3C). Lastly, the capacity to heal a scratch wound was not significantly affected by B4GALNT2 expression clones (Figure 3D).

### 3.3. B4GALNT2 Expression Reduces the Number of Cancer Stem Cells

To investigate the relationship between B4GALNT2 expression and stemness, we analyzed the three cell lines for the expression of aldehyde dehydrogenase (ALDH), reported to be a stem-cell and cancer-initiating cell marker in many tissues, including colon tissue [20].

**Figure 4:**
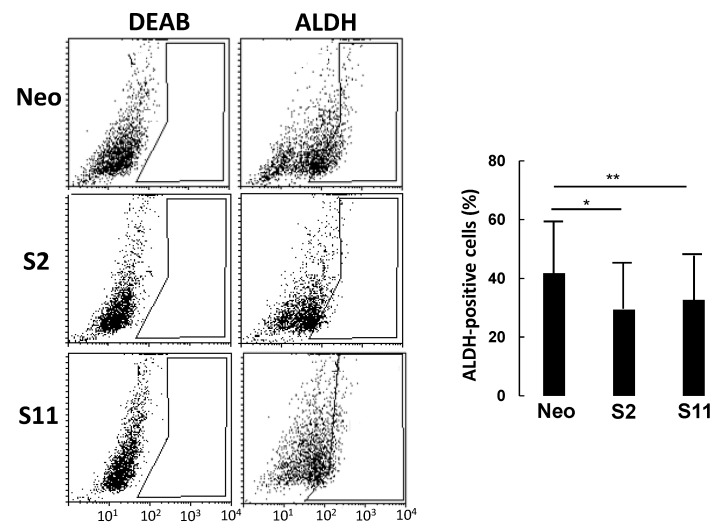
ALDEFLUOR analysis. Cells were incubated with ALDEFLUOR either in the presence or in the absence of the inhibitor DEAB. Gates excluding all of the cells labelled in the presence of DEAB were set. Cells included in the gate in the absence of DEAB were considered to be ALDH-positive. Histograms report the percentage of ALDH-positive cells ± SD in three independent experiments. * *p* ≤ 0.05, ** *p* ≤ 0.01.

In a typical experiment (Figure 4), cells were incubated with the ALDH substrate ALDEFLUOR, either in the presence or in the absence of DEAB (a specific ALDH inhibitor) to provide a negative control. While the percentage of ALDH-positive cells in LS174T Neo was about 40%, it was around 30% in the two B4GALNT2 clones, consistent with a marked reduction in the number of cancer stem cells (CSC).

### 3.4. Impact of B4GALNT2 Expression on the Transcriptome of Colon Cancer Cells

**Figure 5:**
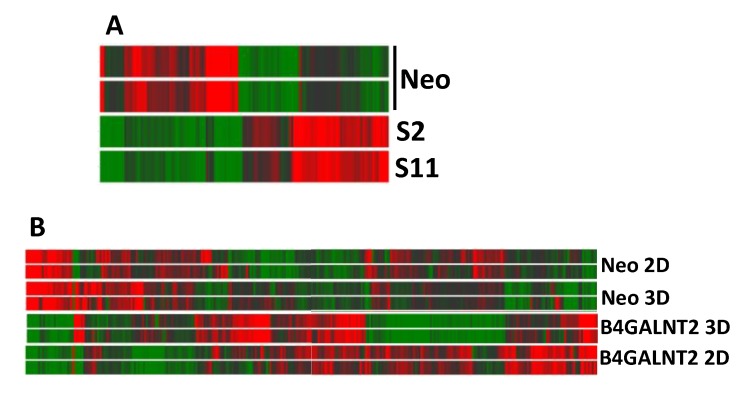
Heatmaps of gene expression analysis. (**A**) B4GALNT2-expressing and control Neo LS174T cells grown in standard 2D conditions. (**B**) Cells grown in 3D conditions or in standard 2D conditions. High and low expression was normalized to the average expression across all samples. Differences were analyzed by the moderated *t*-test. Corrected *p*-value cut-off: 0.15; multiple test correction used: Benjamini–Hochberg.

To understand the origin of the dramatic effect of B4GALNT2 on the phenotype of LS174T cells and, in particular, on the ability to grow in non-adherent conditions, the impact of B4GALNT2 and of 3D growth in liquid medium on the transcriptome of LS174T cells was investigated by microarray analysis. Using this technology, the mean level of B4GALNT2 expression was found to be three in Neo and 230 in S2/S11 cells. The heat map graph displayed in Figure 5A reports the modulation of 142 genes showing a fold change ≥ 2 in LS174T S2 and S11, compared with Neo cells, grown in standard conditions.

Table 2 shows a synthesis of pathway analysis, indicating that stemness, intracellular signaling, and cell adhesion were among the significantly affected pathways.

A more in-depth analysis focused on the 25 genes modulated by B4GALNT2 by a fold change ≥ 4 (Table 3), revealed that four genes displayed up-regulation (above the red line) and 21 displayed down-regulation (below the red line).

A large number of modulated genes were found to be related with cancer. Through an extensive survey of the literature, we attributed a cancer-promoting activity or a cancer-restraining activity to the majority of the modulated genes. A violet or yellow label was assigned on the basis of the putative tumor-promoting or tumor-restraining change (violet for up-regulation of tumor-promoting or down-regulation of tumor-restraining and vice versa for the yellow label). Interestingly, only three changes were putatively tumor-promoting and 12 were tumor-restraining. Some genes, which appeared to be virtually switched off in B4GALNT2-expressing cells, are involved in the basic properties of cancer cells, such as stemness (*SOX2, ROR1*), epithelial to mesenchymal transition (EMT) (*NID1, ALX1*), and growth (*FAM110B, PEG10, MID2*). Overall, the transcriptomic changes induced by B4GALNT2 were consistent with a general down-regulation of gene expression and an attenuation of the neoplastic phenotype.

These data are consistent with a driving role of B4GALNT2 or its cognate Sd^a^ antigen in the regulation of several crucial genes in LS174T cells. If this driving role of B4GALNT2 on gene expression was also exerted in CRC tissues, a consistent regulation of these genes in TCGA patients could be observed. Thus, we asked whether those genes that we found to be up-regulated in LS174T S2 and S11 cells were also up-regulated in patients showing high B4GALNT2 levels in cancer tissues and vice versa for genes displaying down-regulation in S2/S11 cells. To this aim, we considered the same cohorts of patients shown in Figure 2G, comprising 15% of non-expressers and 15% of high expressers. For the 25 genes showing modulation by B4GALNT2 reported in Table 3, we determined the mean level of expression in the non-expressers and in the high-expressers cohorts, respectively (Table 4), from TCGA. The “consistency” column indicates whether the observed difference between non-expressers and high-expressers was consistent with the hypothesized role of B4GALNT2 in regulating gene expression. Out of the 25 genes, one was not expressed and 13 showed a difference consistent with the supposed role of B4GALNT2; for six genes, the change was statistically significant.

### 3.5. B4GALNT2 Expression Regulates the Transcriptional Response to 3D Culture

Owing to the markedly reduced ability to adapt to non-adherent growth displayed by B4GALNT2-expressing cells, we asked which genes were modulated by 3D culture in LS174T cells and which genes displayed a differential response to 3D culture conditions in B4GALNT2-expressing cells S2/S11. Many genes were modulated by 3D culture conditions, regardless of B4GALNT2 expression (Figure 5B). Among these, 106 displayed a fold change ≥ 4 (Table S2). Several strongly modulated genes were involved in energy metabolism, including glycolysis (*PFKFB4, ALDOC, PGM1, PGK1*), and several were part of the hypoxia response (*CA9, EGLN3, EGR1*). Some of these genes displayed extremely high expression levels and were all up-regulated (*PGK1, LCN15, FABP1, ALDOC, PGM1, CA9*). Among the genes involved in transcriptional regulation, the high level of expression of transcription factors *FOS* and *FOSB* and of the transcriptional regulator *EGR1* in 2D culture and their dramatic down-regulation in 3D culture is noteworthy. On the other hand, among the genes involved in cell signaling, the gene *KIT* encoding a crucial tyrosine kinase receptor displayed a high level of up-regulation in 3D culture. Genes involved in cytoskeleton organization exhibited a general down-regulation, while those involved in detoxification displayed up-regulation. Finally, the strong up-regulation of the genes *PIGZ*, involved in the biosynthesis of the glycosylphosphatidylinositol (GPI)-anchor, and *NDRG1*, involved in stress response is of note.

All the above-mentioned genes displayed 3D culture modulation regardless of B4GALNT2 expression. A search for genes differentially modulated by 3D culture depending on the expression or non-expression of B4GALNT2 yielded a list of 31 genes, 13 of which showed up-regulation in response to 3D culture only in S2/S11 cells, while the remaining 18 showed down-regulation in response to 3D culture only in S2/S11 cells (Table 5). The most remarkable change is the concomitant strong down-regulation of five genes (*KIZ, CEP120, DNAH6, SGOL2, STARD13*) controlling cytoskeletal organization in mitosis and motility. This finding could provide a clue to explain the reduced ability of the B4GALNT2-expressing cells to adapt to non-adherent conditions of growth. Among the genes involved in cell signaling, it is important to highlight the concomitant down-regulation of three genes encoding taste receptors, which, besides tasting bitterness, can play a tumor-suppressive role. 3D culture appears to increase the propensity to apoptosis in B4GALNT2-expressing cells by modulating at least three genes: *TNFAIP8L2, MYOD1*, and *PPM1K*. Among the genes involved in transcriptional regulation, the marked down-regulation of *PHF20L1*, which stabilizes SOX2 post-translationally [21], must be noted, and is consistent with reduced stemness. Of the three genes related to immunity and inflammation, *CTLA4* is a well-known inhibitory receptor of T lymphocytes and is surprisingly expressed by this CRC cell line. *IL1A* is an inflammatory cytokine, while *TDO2* is involved in a pathway potentially suppressing anti-tumor immune responses.

Afterward, we investigated whether the TCGA cohorts of non- or high B4GALNT2 expressers displayed parallel modulation of the same genes, focusing on genes belonging to the major functional classes (top five classes of Table 5). Table 6 shows that genes belonging to the groups “Cytoskeleton and mitosis” and “Transcription regulation” in Table 5 displayed significant consistent modulation in TCGA data, but genes *TNFAIP8L2*, involved in apoptosis, and *IL1A* involved in inflammation and immunity, were also consistently modulated. Altogether, these data point to some mechanisms through which B4GALNT2 expression reduces the ability to grow in conditions of poor adherence.

## 4. Discussion

In this study, we show for the first time that in a large cohort of colon cancer samples, the mRNA of *B4GALNT2* is dramatically down-regulated compared with normal tissue, consistent with previous observations of a reduced B4GALNT2 enzymatic activity in CRC tissues [8,10]. In addition, we observed that all patients surviving beyond three years displayed a high level of B4GALNT2 mRNA, indicating a strong relationship between high B4GALNT2 and lower malignancy. In other TCGA cohorts, the association of high B4GALNT2 with survival was not observed, suggesting that it is restricted to CRC. To understand whether high B4GALNT2 and lower malignancy were causally related, we analyzed the phenotype of LS174T cells permanently modified to express B4GALNT2 cDNA. This cell line was chosen because it is devoid of B4GALNT2 activity and of cognate Sd^a^ antigen (like the vast majority of colon cancer cell lines) and is one of the few CRC cell lines expressing the sLe^x^ antigen. In our system, B4GALNT2 expression resulted in a partial replacement of the sLe^x^ epitope with Sd^a^. While little or no effect was observed on the ability to heal a scratch wound or to form clones in standard conditions of growth, B4GALNT2 expression resulted in a dramatic inhibition of the ability to grow in poor or no adherence, pointing to a specific effect of B4GALNT2 in regulating this property. The ability to survive and proliferate without the intracellular signals generated by the mechanosensors [23] is intimately associated with resistance to apoptosis and stemness. Consistently, we observed that in B4GALNT2-expressing cells, the percentage of stem cells was reduced. For this reason, we investigated the effect of B4GALNT2 on the transcriptome of transfected LS174T cell variants. The ability to modulate the transcriptome of cancer cells by a glycosyltransferase was previously documented [17,24,25,26]. Nevertheless, the transcriptomic changes induced by B4GALNT2 in standard conditions of growth were surprisingly relevant. In particular, the marked transcriptional down-regulation of protein-coding genes in B4GALNT2-expressing cells may conceivably be related to a reduced ability to perform several cellular functions. The fact that the vast majority of these genes displayed cancer-promoting activity in different systems is consistent with an attenuation of the cancer phenotype in LS174T cells. Moreover, a causative role of B4GALNT2 in the control of these genes was supported by the observation that in the TCGA cohorts of high and low B4GALNT2 expressers, the mean level of expression of these genes was in some cases significantly consistent with that observed in LS174T cells. Among the genes showing significant inverse association with B4GALNT2 expression in TCGA, *SOX2* is particularly relevant for its role in CRC stemness [27]. Its dramatic down-regulation in S2/S11 clones can certainly contribute to explaining their reduced ability to grow in non-adherent conditions and their reduced content of stem cells. To understand which genes were modulated by non-adherent growth, we analyzed the transcriptome of cells grown as spheroids in 3D conditions. We were particularly interested in understanding which genes were differentially modulated by 3D growth, depending on their B4GALNT2 expression. Growth in 3D conditions modulates a large number of genes, mainly involved in energy metabolisms, transcription, cell signaling, and detoxifying activity. Several genes belong to the hypoxia response, which is not surprising, considering the hypoxic conditions present at the center of the spheroids [28]. However, the genes responding to 3D growth only in B4GALNT2-expressing cells appeared to impair mainly the function of microtubules in mitosis but also transcription, cell signaling, and apoptosis, thus explaining the reduced ability of B4GALNT2-expressing cells to grow in poorly adherent conditions. Interestingly, all the genes belonging to the groups “Cytoskeleton and mitosis” and “Transcription regulation” displayed consistent association with B4GALNT2 level in TCGA cohort. In addition, the gene *TNFAIPL2,* which promotes apoptosis, and the gene of the pro-inflammatory cytokine, *IL1A,* also displayed consistent modulation in the TCGA cohort. Although IL1A plays different and sometimes opposite roles when expressed by cells of the tumor microenvironment, when it is expressed by CRC cells, it is immunostimulatory and induces an anti-tumor immune response [29]. Current data support the notion that high B4GALNT2 is causally related to a better prognosis because it can reduce stemness and capability to grow in poorly adherent conditions, and it increases the propensity to apoptosis and stimulates anti-cancer immunity. Although our data do not clarify whether the observed effects of B4GALNT2 expression are due to the de novo expression of the Sd^a^ antigen or the down-regulation of sLe^x^, they point to the potential usefulness of B4GALNT2 to stratify patients’ risk, contributing to the design of a personalized therapy of colon cancer.

## Figures and Tables

**Table 1:** Genes modulated in high/low B4GALNT2 cohorts.

Genes Up-Regulated in High B4GALNT2 Expressers				
Gene Name	Protein	Functional Class	Functions	*p*-Value
*CLCA1*	Chloride Channel Accessory 1	Mucin-related	Involved in chloride conductance and regulation of mucus production. Potential tumor suppressor. Induces MUC5AC.	****
*FCGBP*	Fc Fragment of IgG Binding Protein		May be involved in the maintenance of the mucosal structure as a gel-like component of the mucosa.	****
*MUC2*	Mucin 2, Oligomeric Mucus/Gel-Forming		Coats the epithelia of the intestines and other mucous membranes. Downreguled in inflammatory bowel diseases.	****
*AGR2*	Anterior Gradient 2, Protein Disulphide Isomerase Family Member		Required for MUC2 post-transcriptional synthesis and secretion. Proto-oncogene.	****
*MUC5B*	Mucin 5B, Oligomeric Mucus/Gel-Forming		Coats the epithelia of the mucous membranes.	**
*LCN2*	Lipocalin 2	Immune function	Limits bacterial growth by sequestering iron-containing siderophores.	*
*IGJ*	Joining Chain Of Multimeric IgA And IgM		Joins IgM and IgA in multimeric complexeses.	****
*PIGR*	Poly-immunoglobulin receptor		Allows the trans-epithelial transport of polymeric IgA to the gut lumen.	****
*REG1A*	Regenerating family member 1α	Regeneration and repair	Regenerating proteins are acute phase reactants, lectins, antiapoptotic or growth factors.	****
*REG4*	Regenerating family member 4			****
*TFF3*	Trefoil factor 3		Involved in maintenance and repair of the intestinal mucosa. Promotes the mobility of epithelial cells in healing processes.	****
*SERPINA1*	Serpin family A member 1	Protease inhibition	Serine protease inhibitor for elastase, plasmin, thrombin, trypsin, chymotrypsin, and plasminogen activator.	***
*SPINK4*	Serine Peptidase Inhibitor Kazal Type 4		Serine-type endopeptidase inhibitor activity.	*
*ITM2C*	Integral Membrane Protein 2C		Secretase inhibitor.	**
*LGALS4*	Galectin 4	Binding of glycoconjugates	Expression restricted to small intestine, colon, and rectum, and it is down-regulated in colorectal cancer.	****
**Genes Up-Regulated in Low B4GALNT2 Expressers**				
*IGF2*	Insulin Like Growth Factor 2	Growth factor	Growth-promoting activity.	****

Genes showing a percentage change (PC) higher than 50 were identified according to the formula: PC = (Mean 15% high − Mean 15% low)/Mean All x 100. Bonferroni’s multiple comparisons test was used for the comparison between the two cohorts. * *p* ≤ 0.05, ** *p* ≤ 0.01, *** *p* ≤ 0.001, **** *p* ≤ 0.0001.

**Table 2:** Pathways modulated by B4GALNT2 expression.

Pathway Map	Network Objects
Stem cell pathways	SOX2, FGFR3, HEY2, IGF1, c-Kit, MEF2C, MLRC, MyHc
Blood coagulation	MyHC, Coagulation factor V, PAR1
Main growth factor signaling cascades	FGFR3, IGF-1
Chemoresistance pathways	c-Kit, IGF-1
Cell adhesion	Nidogen, IGF-1, MyHC, MRLC
Cytoskeleton remodeling	MyHC, MRLC
G protein-coupled receptors signaling	Gα(i)-specific peptide GPCRs, Gα(q)-specific peptide GPCRs

The analysis of differentially expressed genes was determined using the web-based software MetaCore from GeneGo (Thomson Reuters). Original analysis data are reported in Table S1.

**Table 3:** Genes highly modulated by B4GALNT2 in LS174T cells.

Gene Symbol	Expression		Fold Change S2/S11 Vs. Neo	*p*-Value S2/S11 Vs. Neo	Gene Name	Function in Cancer	PMID	
	Neo	S2/S11						
*CD200*	2	27.0	16.8	0.0411	CD200 molecule	Possible colon cancer stem cell marker	27574016	
*NGFRAP1*	39	456.3	11.6	0.0383	Nerve growth factor receptor (TNFRSF16) associated protein 1	Overexpression inhibits growth of breast tumor xenografts.	26408910	
*SKAP1*	138	912.3	6.6	0.0231	Src kinase associated phosphoprotein 1	Modulates TCR signaling.	18320039	
*SLC14A1*	2	10.8	5.2	0.0360	Solute carrier family 14 (urea transporter), member 1 (Kidd blood group)	Potential tumor suppressor in lung cancer	22223368	
*FAM26F*	8	2.0	−4.1	0.0195	Family with sequence similarity 26, member F	Little or no information		
*FAM110B*	9	2.1	−4.5	0.0142	Family with sequence similarity 110, member B	Promotes growth of prostate cancer cells	21919029	
*ALX1*	12	2.6	−4.6	0.0167	ALX homeobox 1	Promotes EMT and invasion in ovarian and lung cancer.	26722397 23288509	
*F5*	12	2.6	−4.7	0.0331	Coagulation factor V (proaccelerin, labile factor)	Little or no information		
*INMT*	9	1.8	−4.7	0.0142	Indolethylamine N-methyltransferase	Negatively associated with prostate cancer progression	22075945	
*MYH3*	1198	238.9	−5.0	0.0167	Myosin, heavy chain 3, skeletal muscle, embryonic	Little or no information		
*MBOAT2*	14	2.5	−5.4	0.0383	Membrane bound O-acyltransferase domain containing 2	Little or no information		
*ROR1*	12	1.8	−6.4	0.0163	Receptor tyrosine kinase-like orphan receptor 1	Associated with ovarian cancer stem cells	25411317	
*RAI14*	51	7.7	−6.6	0.0190	Retinoic acid induced 14	Overexpressed in gastric cancer, associated with worse prognosis.	29654694	
*FMO3*	14	1.8	−7.7	0.0253	Flavin containing monooxygenase 3	Involved in de-toxification of drugs.	16800822	
*PEG10*	44	5.3	−8.4	0.0233	Paternally expressed 10	Enhances cell invasion by upregulating β-catenin, MMP-2 and MMP-9	25199998	
*NINL*	244	28.4	−8.6	0.0339	Ninein-like	High expression associates with poor prognosis in prostate cancer	30637711	
*ARMC4*	15	1.7	−8.7	0.0196	Armadillo repeat containing 4	Can be mutated in gastric cancer.	26330360	
*MID2*	32	2.1	−15.0	0.0152	Midline 2	In breast cancer associates with BRCA1 and promotes growth.	26791755	
*SOX2*	28	1.7	−16.5	0.0163	SRY (sex determining region Y)-box 2	Associated with motility and a cancer stem cell phenotype in CRC	29228716 30518951	
*LGALS2*	362	21.5	−16.8	0.0142	Lectin, galactoside-binding, soluble, 2	Elevated in plasma of CRC patients. Promotes adhesion to endothelia.	21933892	
*NPTX1*	42	2.4	−17.3	0.0123	Neuronal pentraxin I	Anti proliferative in colon cancer	29345391	
*GALC*	49	2.0	−24.9	0.0077	Galactosylceramidase	Unclear		
*STARD3NL*	98	3.6	−27.4	0.0346	STARD3 N-terminal like	Little or no information		
*ZNF22*	83	1.9	−44.6	0.0077	Zinc finger protein 22	Little or no information		
*NID1*	459	5.1	−89.4	0.0306	nidogen 1	Promotes EMT and metastasis in ovarian, breast and lung cancer.	28416770 28827399	

The corrected *p*-value was calculated using the multiple test correction Benjamini–Hochberg. *p* < 0.05, fold change mean S2/S11 B4GALNT2 vs. Neo ≥ 4. The red line separates up-regulated genes from down-regulated genes. The violet or yellow labels indicate putative tumor-promoting or tumor-restraining changes, respectively.

**Table 4:** Gene expression comparison between TCGA cohort (Non- and High B4GALNT2 expressers and microarray analyisis of LS174T cells (S2/S11 comparison with Neo).

	Gene Name	Non-B4GALNT2 Expressers	High B4GALNT2 Expressers	Consistency	*p*-Value
		Mean ± SD	Mean ± SD		
**Genes Down Up-Regulated in LS174T S2/S11**	***CD200***	**171 ± 144**	**250 ± 236**	**Yes**	**≤0.01**
	*NGFRAP1*	850 ± 641	843 ± 5655	Yes	N.S.
	*SKAP1*	220 ± 176	205 ± 192	No	
	*SLC4A1*	Not expressed	Not expressed		
**Genes Down-Regulated in LS174T S2/S11**	*FAM110B*	37 ± 33	35 ± 42	Yes	N.S.
	*ALX1*	3 ± 13	4 ± 8	No	
	*F5*	325 ± 968	180 ± 627	Yes	N.S.
	*INMT*	132 ± 153	118 ± 120	Yes	N.S.
	***MYH3***	**41 ± 125**	**16 ± 16**	**Yes**	**≤0.05**
	*MBOAT2*	498 ± 282	660 ± 405	No	
	*ROR1*	27 ± 39	25 ± 31	No	
	*RAI14*	765 ± 420	661 ± 586	Yes	N.S.
	*FMO3*	86 ± 620	26 ± 25	Yes	N.S.
	*PEG10*	136 ± 401	245 ± 685	No	
	***NINL***	**168 ± 203**	**119 ± 123**	**Yes**	**≤0.05**
	*ARMC4*	4 ± 13	6 ± 8	No	
	*MID2*	146 ± 122	150 ± 104	No	
	***SOX2***	**107 ± 281**	**32 ± 117**	**Yes**	**≤0.01**
	*LGALS2*	77 ± 141	128 ± 210	No	
	***NPTX1***	**39 ± 95**	**15 ± 29**	**Yes**	**≤0.01**
	*GALC*	644 ± 525	663 ± 503	No	
	***STARD3NL***	**697 ± 255**	**646 ± 253**	**Yes**	**≤0.1**
	*ZNF22*	625 ± 239	638 ± 267	No	
	*NID1*	1558 ± 993	1544 ± 1496	Yes	N.S.

The cohorts of non-expressers (Mean ± SD = 0 ± 0) and of high-expressers (Mean ± SD = 367 ± 69) represent the 15% lower and higher percentiles of the TCGA cohort. The column “Consistency” indicates whether the difference in gene expression of non- or high B4GALNT2 expressers was consistent with that observed by microarray analysis of our LS174T model. Genes showing statistically significant consistent difference are indicated in bold (*p* ≤ 0.05 Student’s t test for independent samples). N.S. = non-significant.

**Table 5:** Genes highly modulated by 3D only in B4GALNT2-expressing LS174T cells (clones S2/S11).

Gene Symbol	Expression Neo		Expression S2/S11		Fold Change 3D/2D Neo	Fold Change 3D/2D S2/S11	Corrected *p*-Value	Gene Name	Role	Broad Functional Category
	2D	3D	2D	3D						
*KIZ*	16.2	13.4	19.0	3.8	−1.2	−4.9	0.0086	Kizuna centrosomal protein	Centrosomal protein necessary to endure the forces converging on the centrosomes during spindle formation.	Cytoskeleton and mitosis
*CEP120*	7.3	7.5	15.8	3.1	1.0	−5.2	0.0086	Centrosomal protein 120kDa	Functions in the microtubule-dependent coupling of the nucleus and the centrosome.	
*DNAH6*	6.3	5.3	13.1	2.3	−1.2	−5.7	0.0163	Dynein, axonemal, heavy chain 6	Member of the dynein family, which are constituents of the microtubule-associated motor protein complex.	
*SGOL2*	8.6	7.7	18.4	2.2	−1.1	−8.3	0.0086	Shugoshin-like 2 (S. pombe)	Targets PPP2CA to centromeres, leading to cohesin dephosphorylation.	
*STARD13*	13.5	16.3	19.8	4.2	1.2	−4.7	0.0156	StAR-related lipid transfer (START) domain containing 13	Involved in regulation of cytoskeletal reorganization, cell proliferation and motility.	
*UPK1A*	4.0	3.7	1.9	12.0	−1.1	6.3	0.0086	Uroplakin 1A	Member of the tetraspanin family, mediates signaling. Decreased expression is associated with CRC progression and poor prognosis. (PMID: 25197375)	Cell signaling
*OR52R1*	11.2	10.3	3.3	15.5	−1.1	4.8	0.0131	Olfactory receptor, family 52, subfamily R, member 1 (gene/pseudogene)	Olfactory receptors are G-protein-coupled receptors involved in perception of smell and other functions.	
*TAS2R45*	42.6	35.9	68.8	17.2	−1.2	−4.0	0.0247	Taste receptor, type 2, member 45	Taste receptors play a role in the perception of bitterness and in sensing the chemical composition of the gastrointestinal content. Some taste receptors inhibit cancer growth and stemness. (PMID: 28467517)	
*TAS2R19*	41.9	31.8	70.3	17.1	−1.3	−4.1	0.0116	Taste receptor, type 2, member 19		
*TAS2R30*	254.5	215.3	402.3	81.8	−1.2	−4.9	0.0168	Taste receptor, type 2, member 30		
*TNFAIP8L2*	4.7	6.0	2.0	14.5	1.3	7.3	0.0319	Tumor necrosis factor, alpha-induced protein 8-like 2	Promotes Fas-induced apoptosis. (PMID: 28186089)	Apoptosis
*MYOD1*	3.4	5.7	2.3	10.4	1.7	4.4	0.0239	Myogenic differentiation 1	Mediates apoptosis through caspase 3. (PMID: 28131747)	
*PPM1K*	16.3	11.7	25.7	4.0	−1.4	−6.5	0.0086	Protein phosphatase, Mg2+/Mn2+ dependent, 1K	Regulates the mitochondrial permeability transition pore and is essential for cellular survival.	
*SDPR (CAVIN2)*	4.3	1.8	18.3	2.3	−2.4	−8.1	0.0106	Serum deprivation response	Role in caveolar biogenesis and morphology. Metastasis suppressor and activator of apoptosis. (PMID: 26739564).	
*PHF20L1*	7.4	7.1	24.2	2.7	1.0	−8.9	0.0089	PHD finger protein 20-like 1	Predicted to be involved in regulation of transcription. Stabilizes SOX2 postranslationally. (PMID: 30089852)	Transcription regulation
*KLF12*	5.2	3.9	12.3	2.2	−1.3	−5.7	0.0235	Kruppel-like factor 12	Inhibitor of the AP-2 alpha transcription factor. Inhibits growth and anoikis resistance of ovarian cancer cells. (PMID: 28095864)	
*PCF11*	3.6	5.0	6.7	1.7	1.4	−4.0	0.0086	PCF11 cleavage and polyadenylation factor subunit	It is necessary for efficient Pol II transcription termination	
*CTLA4*	2.1	3.7	2.4	14.2	1.7	5.8	0.0136	Cytotoxic T-lymphocyte-associated protein 4	Inhibitor of T cell activation.	Immunity and inflammation
*IL1A*	1.7	2.8	2.1	11.7	1.7	5.6	0.0259	Interleukin 1α	Involved in immune responses and inflammatory processes.	
*TDO2*	3.1	6.5	7.3	29.0	2.1	4.0	0.0086	Tryptophan 2,3-dioxygenase	In tryptophan metabolism catalyzes the first step of the kynurenine pathway. Increased kynurenine may suppress antitumor immune responses.	
*FSIP2*	8.3	5.8	17.0	2.9	−1.4	−5.9	0.0365	Fibrous sheath interacting protein 2	Protein associated with the sperm fibrous sheath.	Fertilization
*SPACA1*	5.5	6.4	4.1	18.2	1.2	4.5	0.0293	Sperm acrosome associated 1	Localizes to the acrosomal membrane of spermatozoa, playing a role in acrosomal morphogenesis and in sperm-egg fusion.	
*USP11*	6.1	7.0	3.3	13.3	1.1	4.0	0.0352	Ubiquitin specific peptidase 11	Encodes a cysteine protease that cleaves ubiquitin from ubiquitin-conjugated protein substrates.	Ubiquitination
*ST13*	6.9	12.8	2.7	11.0	1.9	4.1	0.0090	Suppression of tumorigenicity 13 (colon carcinoma) (Hsp70 interacting protein)	Mediates the association of the heat shock proteins HSP70 and HSP90.	Protein folding
*HIST4H4*	81.2	193.8	101.6	406.8	2.4	4.0	0.0365	Histone cluster 4, H4	Component of the nucleosome.	Chromatin structure
*TRAPPC2*	10.3	7.1	8.4	1.9	−1.5	−4.4	0.0086	Trafficking protein particle complex 2	May play a role in vesicular transport from endoplasmic reticulum to Golgi	Intracellular transport
*C8orf74*	6.3	5.3	10.1	2.3	−1.2	−4.4	0.0135	Chromosome 8 open reading frame 74	Little or no information	
*NAALADL2*	15.5	10.4	17.9	4.1	−1.5	−4.3	0.0196	N-acetylated alpha-linked acidic dipeptidase-like 2		
*SAMD12*	21.1	17.1	25.8	6.3	−1.2	−4.1	0.0323	Sterile alpha motif domain containing 12		
*FRG2*	40.8	49.1	40.2	173.8	1.2	4.3	0.0138	FSHD region gene 2		
*FRG2C*	3.5	8.4	7.2	35.0	2.4	4.9	0.0124	FSHD region gene 2 family, member C		

Corrected *p*-value was calculated using the multiple test correction Benjamini–Hochberg (*p* < 0.05, fold change B4GALNT2 3D vs. B4GALNT2 2D). Information on the gene role were obtained from Genecards [22] and from PubMed.

**Table 6:** Gene expression comparison between TCGA cohort (Non- and High B4GALNT2 expressers and microarray analyisis of LS174T cells (modulated by 3D only in S2/S11).

	Gene Name	Non-B4GALNT2 Expressers	High B4GALNT2 Expressers	Consistency	*p*-Value
		Mean ± SD	Mean ± SD		
Cytoskeleton and mitosis	***KIZ (PLK1S1)***	**346 ± 302**	**250 ± 154**	**Yes**	**≤0.01**
	***CEP120***	**404 ± 116**	**340 ± 135**	**Yes**	**≤0.01**
	***DNAH6***	**33 ± 28**	**27 ± 15**	**Yes**	**≤0.05**
	***SGOL2***	**316 ± 124**	**273 ± 138**	**Yes**	**≤0.01**
	***STARD13***	**529 ± 271**	**355 ± 211**	**Yes**	**≤0.01**
Cell signaling	*UPK1A*	16 ± 112	5 ± 21	Yes	**N.S.**
Apoptosis	***TNFAIP8L2***	**46 ± 38**	**58 ± 42**	**Yes**	**≤0.05**
	*PPM1K*	97 ± 133	10 ± 45	No	
	*SDPR*	186 ± 271	153 ± 162	Yes	N.S
Transcription regulation	***PHF20L1***	**941 ± 296**	**794 ± 349**	**Yes**	**≤0.01**
	***KLF12***	**239 ± 199**	**179 ± 128**	**Yes**	**≤0.01**
	***PCF11***	**1025 ± 302**	**856 ± 227**	**Yes**	**≤0.01**
Immunity and inflammation	*CTLA4*	47 ± 85	42 ± 34	No	
	***IL1A***	**34 ± 86**	**69 ± 215**	**Yes**	**≤0.05**
	*TDO2*	232 ± 885	115 ± 156	No	

The mean level of expression in TCGA database of genes selectively modulated by 3D growth only in S2/S11 cells (Table 5) was compared in the cohorts of non-B4GALNT2 expressers and of high B4GALNT2 expressers as in Table 4. The column “Consistency” indicates whether the difference observed in the cohorts was consistent with that reported in Table 5. Genes showing statistically significant consistent difference are indicated in bold (*p* ≤ 0.05 Student’s t test for independent samples). N.S. = non-significant. A few genes present in Table 5 are not present in this Table because they were not present in TCGA or not expressed.

## Supplementary Materials

The following are available online at https://www.mdpi.com/2073-4409/9/4/948/s1: Table S1: Pathways modulated by B4GALNT2 expression; Table S2: Genes highly modulated by 3D culture in LS174T.
